# Bergenin Reduces Experimental Painful Diabetic Neuropathy by Restoring Redox and Immune Homeostasis in the Nervous System

**DOI:** 10.3390/ijms21144850

**Published:** 2020-07-09

**Authors:** Cristiane F. Villarreal, Dourivaldo S. Santos, Pedro S. S. Lauria, Kelly B. Gama, Renan F. Espírito-Santo, Paulo J. L. Juiz, Clayton Q. Alves, Jorge M. David, Milena B. P. Soares

**Affiliations:** 1 Faculdade de Farmácia, Universidade Federal da Bahia, CEP 40.170-115 Salvador, Brazil; dourivaldo2010@hotmail.com (D.S.S.); pedrosslauria@gmail.com (P.S.S.L.); r.fernandes88@hotmail.com (R.F.E.-S.); 2 Instituto Gonçalo Moniz, Fundação Oswaldo Cruz, CEP 40.296-710 Salvador, Brazil; kellybgama@yahoo.com.br (K.B.G.); milena@cpqgm.fiocruz.br (M.B.P.S.); 3 Universidade Federal do Recôncavo da Bahia, CEP 44.042-280 Feira de Santana, Brazil; paulojuiz@gmail.com; 4 Departamento de Ciências Exatas, Universidade Estadual de Feira de Santana, CEP 44.036-336 Feira de Santana, Brazil; cleiroz@gmail.com; 5 Instituto de Química, Universidade Federal da Bahia, CEP 40.170-280 Salvador, Brazil; jmdavid@ufba.br

**Keywords:** neuropathic pain, diabetic neuropathy, natural product, analgesic, antioxidant, immunomodulation, cytokine, spinal cord, sciatic nerve

## Abstract

Diabetic neuropathy is a frequent complication of diabetes. Symptoms include neuropathic pain and sensory alterations—no effective treatments are currently available. This work characterized the therapeutic effect of bergenin in a mouse (C57/BL6) model of streptozotocin-induced painful diabetic neuropathy. Nociceptive thresholds were assessed by the von Frey test. Cytokines, antioxidant genes, and oxidative stress markers were measured in nervous tissues by ELISA, RT-qPCR, and biochemical analyses. Single (3.125–25 mg/kg) or multiple (25 mg/kg; twice a day for 14 days) treatments with bergenin reduced the behavioral signs of diabetic neuropathy in mice. Bergenin reduced both nitric oxide (NO) production in vitro and malondialdehyde (MDA)/nitrite amounts in vivo. These antioxidant properties can be attributed to the modulation of gene expression by the downregulation of inducible nitric oxide synthase (iNOS) and upregulation of glutathione peroxidase and Nrf2 in the nervous system. Bergenin also modulated the pro- and anti-inflammatory cytokines production in neuropathic mice. The long-lasting antinociceptive effect induced by bergenin in neuropathic mice, was associated with a shift of the cytokine balance toward anti-inflammatory predominance and upregulation of antioxidant pathways, favoring the reestablishment of redox and immune homeostasis in the nervous system. These results point to the therapeutic potential of bergenin in the treatment of painful diabetic neuropathy.

## 1. Introduction

Diabetes is a debilitating metabolic disorder that can lead to several complications, affecting not only patients’ lives, but society as a whole [1]. The global estimate of diabetes prevalence in the year of 2040 is 7.8%, meaning that two decades from now there could be over 480 million cases worldwide [2]. Around 50% of those patients are likely to develop diabetic neuropathy (DN), the most frequent chronic complication of both type 1 and type 2 diabetes [3]. Clinical manifestations of DN include painful neuropathic symptoms, such as experience spontaneous prickling, stabbing or burning sensations, hyperalgesia and allodynia, and sensory loss, resulting in foot ulcerations and amputations [3,4]. The treatment of painful DN remains a challenge. Current strategies rely on the maintenance of blood glucose levels and pharmacological control of pain [5]. Drug classes currently used in DN treatment include tricyclic antidepressants, serotonin and noradrenaline reuptake inhibitors, and gabapentinoids [6]. However, these drugs only promote moderate pain relief and do not elicit disease-modifying effects [6,7]. Because of this gap in the pharmacological control of painful DN, researchers have been looking for more effective treatments. Pre-clinical studies have been shedding light on new therapeutic approaches to painful DN, such as the use of cell therapy [8] and numerous natural compounds [9].

**Figure 1:**
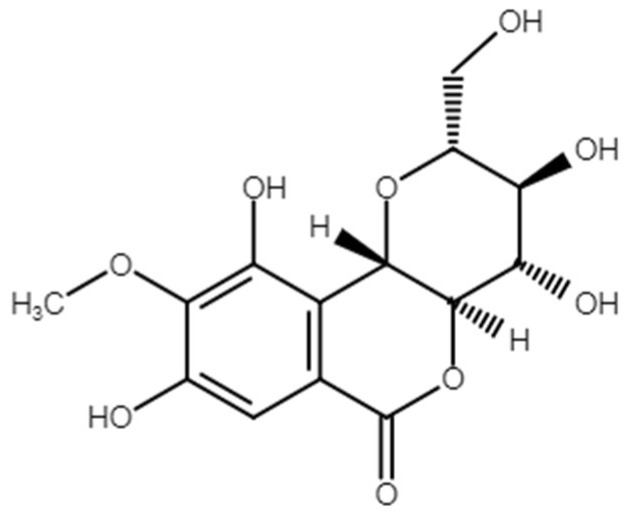
Chemical structure of bergenin.

Bergenin (Figure 1) is a naturally occurring C-glucoside of 4-O-methylgallic acid found in many plant species. Several therapeutic applications of bergenin have been proposed in the literature, e.g., anti-ulcerative [10], hepatoprotective [11,12], anticancer [13], neuroprotective [14,15], antidiabetic [16], anti-inflammatory [17,18,19,20,21,22], antinociceptive [17,23], and antioxidant [12,16,24,25,26,27]. Considering that oxidative stress and neuroinflammation play key roles in the pathophysiology of DN [28], the above-mentioned properties of bergenin may contribute to the effective control of painful DN. The present study was designed to investigate this hypothesis in a preclinical setting, using the streptozotocin (STZ)-induced DN model in mice. STZ has been consistently used in preclinical diabetes research for the induction of murine models of insulin-dependent type 1-like diabetes [29] since its diabetogenic properties were first reported [30].

## 2. Results

### 2.1. Acute Bergenin Treatment Ameliorates Established Painful Diabetic Neuropathy

**Figure 2:**
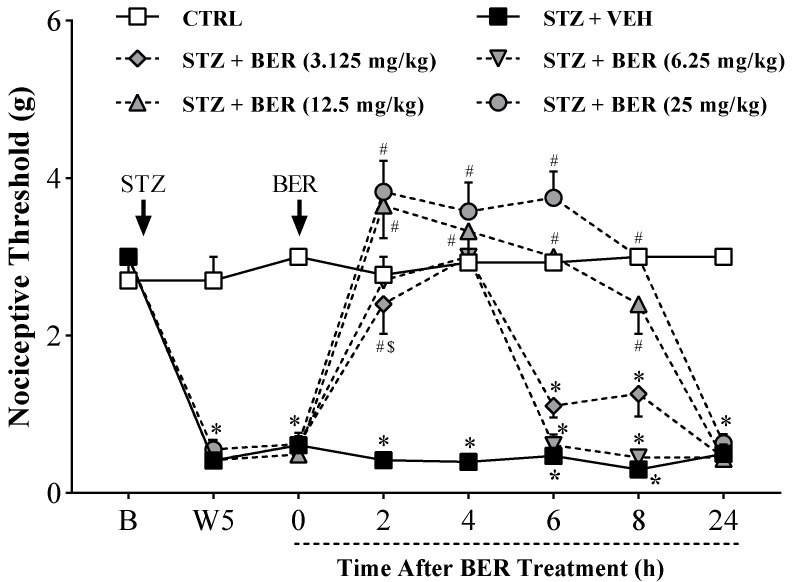
Acute effect of bergenin on diabetic sensory neuropathy. Diabetic mice were intraperitoneally treated with bergenin (BER; 3.125–25 mg/kg) or vehicle (2% dimethyl sulfoxide in saline; VEH). Non-diabetic control group (CTRL) received citrate buffer only. The nociceptive threshold (axis of ordinates) was evaluated prior to the experiment (baseline; B), at the fifth week after streptozotocin (STZ) before treatments (W5), and at different times after bergenin treatment. Values represent the filament weight (g) in which mice responded in 50% of the presentations. Data are expressed as means ± SD (*n* = 6). Two-way ANOVA with repeated measures showed significant difference regarding the time of observation (*p* < 0.05) and treatments (*p* < 0.05), and a significant treatment-time interaction (*p* < 0.05). Bonferroni’s multiple comparisons: * *p* < 0.05 compared with CTRL group. # *p* < 0.05 compared with STZ + VEH group. $ *p* < 0.05 compared with STZ + BER (12.5 mg/kg) and STZ + BER (25 mg/kg) groups.

**Figure 3:**
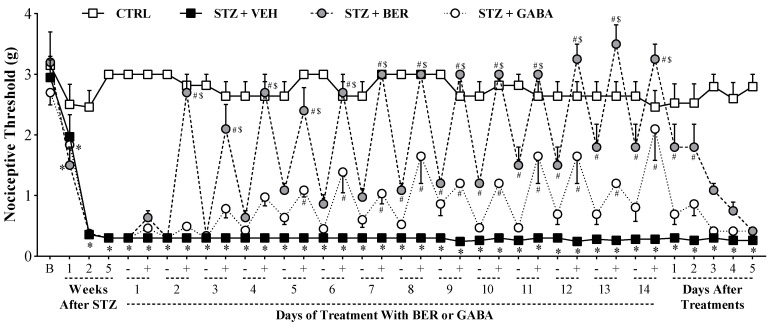
Effect of multiple doses of bergenin on diabetic sensory neuropathy. Diabetic mice were treated with bergenin (BER; 25 mg/kg, i.p.), vehicle (2% dimethyl sulfoxide in saline; VEH, i.p.), or gabapentin (GABA; gold standard drug, 40 mg/kg, p.o.) twice a day for 14 days, starting in the fifth week after STZ. Non-diabetic control group (CTRL) received citrate buffer only. The nociceptive threshold (axis of ordinates) was evaluated prior to the experiment (baseline; B), weekly until the fifth week after STZ, and daily from the beginning of treatments until the last experimental day. During the 14 days of treatment, thresholds were daily assessed in two moments: Immediately before (−) and one hour after (+) the first dose of the day. Values represent the filament weight (g) in which mice responded in 50% of the presentations. Data are expressed as means ± SD (*n* = 6). Two-way ANOVA with repeated measures showed significant difference regarding the time of observation (*p* < 0.05) and treatments (*p* < 0.05), and a significant treatment-time interaction (*p* < 0.05). Bonferroni’s multiple comparisons: * *p* < 0.05 compared with CTRL group. # *p* < 0.05 compared with STZ + VEH group. $ *p* < 0.05 compared with STZ + GABA group.

STZ-induced DN was associated with a severe decrease in mechanical nociceptive threshold (*p* < 0.05), revealing the development of allodynia characteristic of painful neuropathy, which was maintained throughout the experimental period (Figure 2 and Figure 3). The single intraperitoneal administration of bergenin (3.125–25 mg/kg) completely reverted the mechanical allodynia (*p* < 0.05), in a dose-dependent way, in mice with established painful diabetic neuropathy. This antinociceptive effect lasted up to 4 h at the lowest doses and reached 8 h at the highest doses. Neither the treatment with STZ nor with bergenin compromised mice’s motor function in the rota-rod test.

### 2.2. Multiple Doses of Bergenin Revert Behavioral Painful Diabetic Neuropathy

Once the antinociceptive activity of bergenin on sensory neuropathy had been established, and considering that diabetic neuropathy is a chronic syndrome, the effect of multiple doses of bergenin over time was next evaluated (Figure 3). In STZ-treated mice, the decline in nociceptive threshold was significant (*p* < 0.05) in the first week after induction and reached its maximum by the second week, thenceforth remaining in plateau. Intraperitoneal treatment with bergenin at 25 mg/kg every 12 h for 14 days resulted in an increasing antinociceptive effect over time. A consistent reduction of mechanical allodynia was daily observed one hour after bergenin treatment (*p* < 0.05). At first, no residual therapeutic effect was detected when thresholds were assessed daily prior to bergenin treatment. Nevertheless, the prior-to-treatment threshold of bergenin-treated mice gradually increased each day, compared to vehicle-treated mice. This increase was significant (*p* < 0.05) on each day between days 8 and 14 (last day of treatment), suggesting a long-lasting antinociceptive effect of the treatments made in the day before. Daily treatment with gabapentin (40 mg/kg, p.o.), the gold standard drug for clinical control of diabetic neuropathic pain, exhibited antinociceptive effect only in the post-treatment evaluation (*p* < 0.05). However, gabapentin treatment failed to induce the sustained therapeutic effect observed for bergenin. Importantly, bergenin had greater efficacy when compared to gabapentin (*p* < 0.05).

### 2.3. Bergenin Treatment Does Not Reduce Glycemia in Diabetic Mice

**Figure 4:**
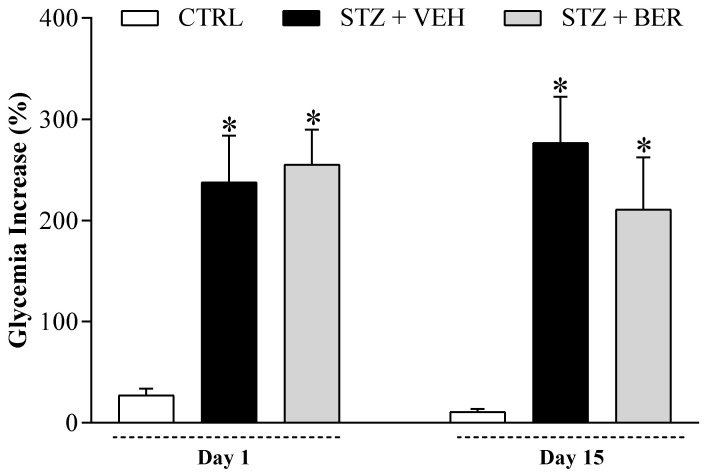
Influence of daily treatment with bergenin on STZ-induced hyperglycemia. Diabetic mice were treated with bergenin (BER; 25 mg/kg, i.p.) or vehicle (2% dimethyl sulfoxide in saline; VEH, i.p.) twice a day for 14 days, starting in the fifth week after STZ. The non-diabetic control group (CTRL) received citrate buffer only. Glycemia levels were assessed prior to the experiment (baseline), after STZ induction and immediately before daily treatments (day 1), and after two weeks of daily treatments (day 15). Glycemia increase (axis of ordinates) was calculated as the percentage rise in blood glucose levels relative to baseline. Data are expressed as means ± SD (*n* = 6). * *p* < 0.05 compared with CTRL group. One-way ANOVA followed by Tukey’s test.

Since one of the ways by which bergenin could have improved painful DN were by modulating diabetes itself, the blood glucose levels of mice were monitored throughout the experimental period (Figure 4). Both groups of diabetic mice had an increase in glycemia following the STZ-induction protocol (*p* < 0.05). Hyperglycemia persisted in both diabetic groups after two weeks of daily treatments with bergenin (25 mg/kg) or vehicle.

### 2.4. Bergenin Reduces NO Production in Vitro

**Figure 5:**
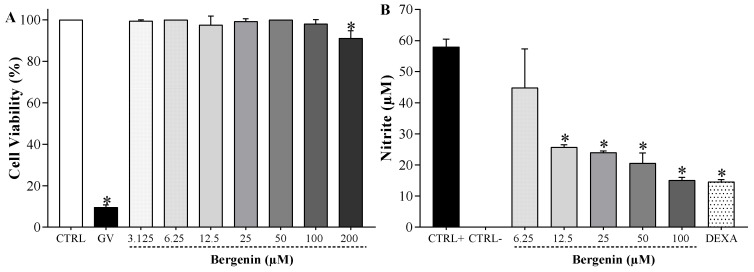
Influence of bergenin on the cell viability and NO production by J774 macrophages. (**A**) Cell viability was determined by the Alamar Blue assay in J774 macrophages incubated for 72 h with bergenin (BER; 3.125–200 μΜ), vehicle (2% dimethyl sulfoxide in saline; CTRL), or gentian violet (GV; 10 μM, positive control). Data are expressed as means ± SD of 3 replicates. * *p* < 0.05 compared with CTRL group. One-way ANOVA followed by Tukey’s test. (**B**) Concentrations of nitrite were determined by the Griess method in J774 macrophages incubated for 24 h with bergenin (BER; 6.25–100 μΜ), vehicle (2% dimethyl sulfoxide in saline; CTRL+), or dexamethasone (DEXA; reference drug, 40 μM) in the presence of LPS (500 ng/mL) and IFN-γ (5 ng/mL). Unstimulated cells were used as negative control (CTRL-). Data are expressed as means ± SD of 3 replicates. * *p* < 0.05 compared with CTRL+ group. One-way ANOVA followed by Tukey’s test.

Considering that oxidative stress plays a key role in the pathophysiology of DN and that previous works have shown the antioxidant potential of bergenin, the hypothesis that antioxidant mechanisms contributed to the therapeutic effect of bergenin in painful DN was next investigated, initially using an in vitro approach. After determining the range of cytotoxic concentration of bergenin to J774 macrophages (Figure 5A), the effect of non-cytotoxic concentrations of this compound on the nitric oxide (NO) production by stimulated J774 cells was evaluated (Figure 5B). Bergenin (12.5–100 μΜ) was able to reduce the amount of nitrite on macrophages stimulated with LPS (500 ng/mL) and IFN-γ (5 ng/mL) in comparison to vehicle-treated stimulated cells (*p* < 0.05), suggesting a reduction of nitric oxide (NO) production. Dexamethasone (40 μM), used as reference drug, also inhibited NO release by stimulated macrophages (*p* < 0.05). These data suggested that bergenin might attenuate pro-oxidative states, which needed confirmation in vivo.

### 2.5. Bergenin Reduces Markers of Oxidative Stress in Nervous System of Neuropathic Mice

**Figure 6:**
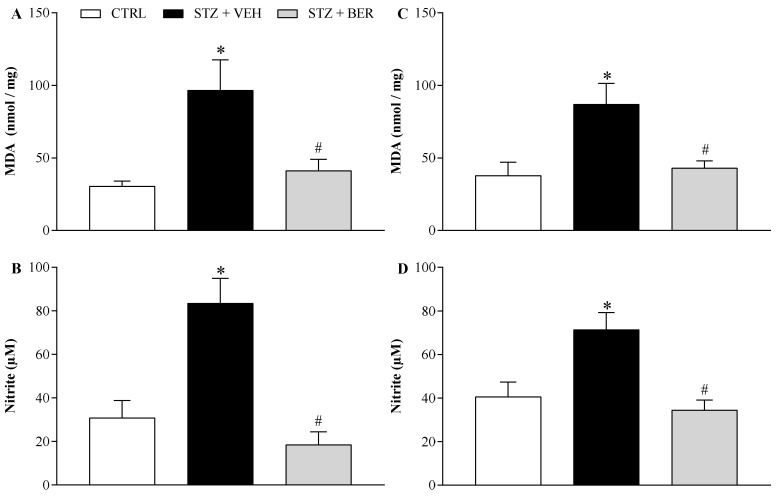
Effect of bergenin on the levels of malondialdehyde (MDA) and nitrite in the nervous system of mice with painful diabetic neuropathy. Diabetic mice were intraperitoneally treated with bergenin (BER; 25 mg/kg) or vehicle (2% dimethyl sulfoxide in saline; VEH) twice a day for 14 days. Non-diabetic control group (CTRL) received citrate buffer only. The levels of MDA and nitrite were measured in the sciatic nerve (**A** and **B**) and spinal cord (**C** and **D**) of mice 24h after the last daily treatment. Data are expressed as means ± SD of 4 replicates. * *p* < 0.05 compared with CTRL group. # *p* < 0.05 compared with STZ + VEH group. One-way ANOVA followed by Tukey’s test.

The effects of bergenin (25 mg/kg, twice a day for 14 days) on lipid peroxidation and nitrosative stress during DN were assessed by measuring the tissue levels of MDA and nitrite, respectively (Figure 6). Painful DN was associated with increased levels of MDA and nitrite in the sciatic nerve (Figure 6A,B; *p* < 0.05) and in the spinal cord (Figure 6C,D; *p* < 0.05). Bergenin treatment effectively reduced (*p* < 0.05) the levels of both biomarkers in both peripheral and central nervous tissues.

### 2.6. Bergenin Modifies the Antioxidant Profile in the Nervous System of Mice with Painful DN

**Figure 7:**
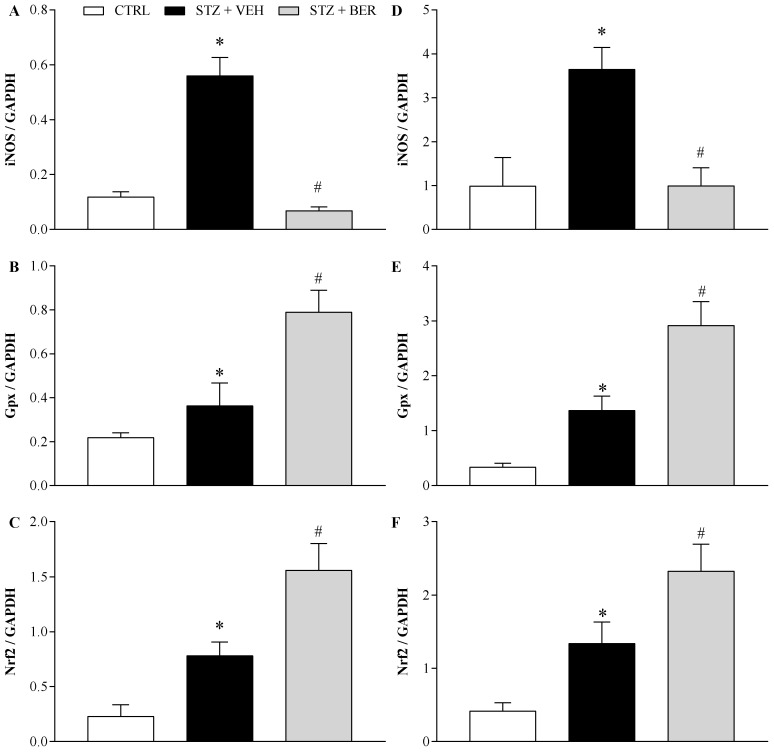
Influence of bergenin on the antioxidant profile in the DRG and spinal cord of mice with painful diabetic neuropathy. Diabetic mice were intraperitoneally treated with bergenin (BER; 25 mg/kg) or vehicle (2% dimethyl sulfoxide in saline; VEH) twice a day for 14 days. Non-diabetic control group (CTRL) received citrate buffer only. The *iNOS*, *Gpx*, and *Nrf2* mRNA levels were measured by RT-qPCR in the DRG (**A**, **B**, and **C**, respectively) and in the spinal cord (**D**, **E**, and **F**, respectively) of mice 24h after the last daily treatment. Data are expressed as means ± SD of 3 replicates. * *p* < 0.05 compared with CTRL group. # *p* < 0.05 compared with STZ + VEH group. One-way ANOVA followed by Tukey’s test.

After confirming that bergenin could modulate oxidative stress in the nervous system during chronic diabetic neuropathy, the next protocol was designed to investigate whether this action was due to the activation of neuroprotective antioxidant pathways. STZ-induced painful DN resulted in increased (*p* < 0.05) mRNA inducible nitric oxide synthase (iNOS) levels in the dorsal root ganglia (DRG; Figure 7A) and spinal cord (Figure 7D). Those levels were drastically reduced by bergenin in both tissues (*p* < 0.05). On the other hand, neuropathic mice showed increased (*p* < 0.05) glutathione peroxidase (*Gpx*; Figure 7B,E) and Nrf2 (Figure 7C,F) mRNA levels in the DRG and in the spinal cord. Daily treatment with bergenin further increased (*p* < 0.05) the mRNA levels of the antioxidant genes, *Gpx* and *Nrf2*, both in DRG and in the spinal cord of mice with painful DN.

### 2.7. Bergenin Modulates Pro- and Anti-Inflammatory Cytokines Production in the Nervous System

**Figure 8:**
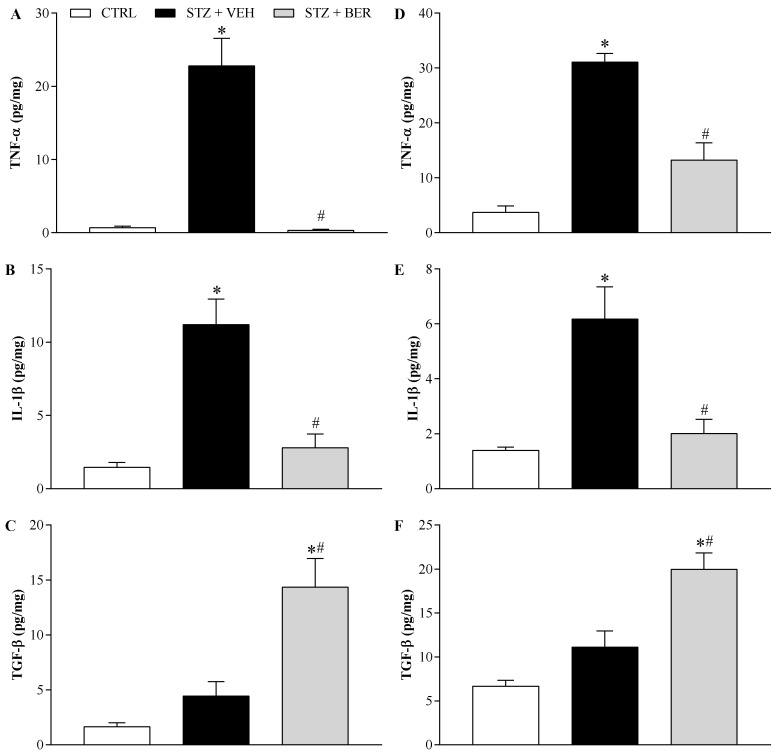
Influence of bergenin on the levels of pro- and anti-inflammatory cytokines in the nervous system of mice with painful diabetic neuropathy. Diabetic mice were intraperitoneally treated with bergenin (BER; 25 mg/kg) or vehicle (2% dimethyl sulfoxide in saline; VEH) twice a day for 14 days. Non-diabetic control group (CTRL) received citrate buffer only. The cytokines tumor necrosis factor-α (TNF-α), interleukin-1β (IL-1β), and transforming growth factor-β (TGF-β) were quantified in the sciatic nerve (**A**, **B,** and **C**, respectively) and spinal cord (**D**, **E,** and **F**, respectively) of mice 24h after the last daily treatment. The results are expressed as picograms of cytokine per milligram of protein. Data are expressed as means ± SD of 3 replicates. * *p* < 0.05 compared with CTRL group. # *p* < 0.05 compared with STZ + VEH group. One-way ANOVA followed by Tukey’s test.

Because the anti-inflammatory activity of bergenin and its ability to modulate the production of cytokines have been previously demonstrated, the influence of bergenin (25 mg/kg, twice a day for 14 days) on the balance of pro- and anti-inflammatory cytokines in the nervous system of DN mice was evaluated (Figure 8). The same modulation profile of bergenin was observed in the sciatic nerve (Figure 8A–C) and in the spinal cord (Figure 8D–F) of mice. In the vehicle-treated group, there was an increase in the pro-inflammatory cytokines tumor necrosis factor-α (TNF-α) and interleukin-1β (IL-1β) levels (*p* < 0.05), whereas the anti-inflammatory cytokine transforming growth factor-β (TGF-β) remained unaltered compared to the control group. In mice daily treated with bergenin, pro-inflammatory cytokines levels were reduced (*p* < 0.05) and TGF-β levels increased significantly (*p* < 0.05).

## 3. Discussion

The therapeutic potential of bergenin in the treatment of diabetes had been previously suggested [16,26,31], though its effect in diabetic neuropathy had not yet been investigated. In the present study, diabetic mice treated with bergenin had a dose-dependent reduction of the behavioral signs of experimental DN. This effect was associated with modulation of the pro- and anti-inflammatory cytokines balance and redox homeostasis in pain pathways in the nervous system. Considering the pivotal role of oxidative stress and neuroinflammation in the pathophysiology of diabetic neuropathy, the properties of bergenin evidenced here highlight the potential of this natural compound for the development of drugs aimed at controlling painful DN.

Sensory neuropathy is a debilitating consequence of diabetes [3]. The currently available treatments have therapeutic limitations, failing to promote satisfying levels of analgesia in patients suffering from DN [6,7]. Herein, the continuous treatment with bergenin for 14 days promoted sustained antinociceptive effect in experimental DN, indicating that this compound did not induce tolerance in mice. The antinociceptive effect was dose-dependent and increased with time, showing a better pharmacological profile than gabapentin, the gold standard drug. Even though Kumar et al. [16] have shown that bergenin reduces the glycemic levels in STZ-induced neuropathic rats, no evidence of hypoglycemic activity of bergenin was found in the present work, which could be explained by the different animal species used in the studies. Moreover, previous works have shown that the treatment of painful DN is not necessarily associated with glycemic control [8,9]. In fact, several complex mechanisms take place in both peripheral and central nervous systems in the genesis of painful DN.

Oxidative stress is believed to be the common denominator of all diabetic complications, with hyperglycemia ultimately leading to reactive oxygen species (ROS) formation and cell damage [4,32]. The oxidation of excessive intracellular glucose via the Krebs cycle results in upregulation of electron transport chain, which exceeds mitochondrial capacity. The surplus of electrons is then transferred to oxygen molecules, generating superoxide (O_2_^-^), which in turn activates damaging pathways and gives rise to other ROS in a chain reaction [32]. Previous studies have shown that bergenin diminishes intracellular levels of superoxide and other ROS by increasing the activity of antioxidant enzymes, such as superoxide dismutase (SOD), catalase (CAT), and glutathione peroxidase (Gpx) [12,16,25,26]. In this work, the antioxidant activity of bergenin was confirmed both in vitro, by the inhibition of NO production by J774 macrophages and in vivo, by the reduction of MDA and nitrite levels in neuropathic mice’s sciatic nerve and spinal cord. Our results are in consonance with those from de Oliveira et al. [24], who demonstrated the preventive action of bergenin against lipid peroxidation and from Wang et al. [33], who showed that bergenin inhibits macrophage activation. The upregulation of iNOS in macrophages has been shown in hyperglycemic obese mice [34]. iNOS is accountable for the production of NO, an important vasoactive mediator which is associated to the processing of inflammatory and neuropathic pain in the spinal cord [35]. Inhibition of iNOS has been associated with the reduction of both mechanical and thermal hyperalgesia in experimental neuropathic pain [36]. In this study, bergenin treatment reduced the levels of iNOS mRNA in neuropathic mice, agreeing with previous reports that bergenin downregulates iNOS [20] and reduces the production of NO [18]. Similar results were found by Ahlawat and Sharma [37], who showed that inhibiting iNOS results in improvement of painful DN in type 2 diabetic rats.

Besides oxidative stress, another consequence of high glucose exposure is the formation of advanced glycation end products (AGEs). These glucose-modified proteins bind to cell surface AGE receptors, initiating inflammatory signaling cascades [32,38]. Cellek et al. [39] demonstrated in STZ-induced diabetic rats that NO acts in a synergistic way with AGEs to increase ROS levels and caspase-3-dependent apoptosis. AGEs and oxidative stress are related to activation of nuclear factor kappa-light-chain-enhancer of activated B cells (NF-ĸB), expression of pro-inflammatory genes, and overproduction of cytokines, which cause further oxidative stress in a positive feedback cycle [32,40]. Nrf2 is a pivotal transcription factor that counteracts the pro-oxidative events [41]. It promotes scavenging of excessive ROS levels [41] and protects mitochondria from oxidant damage [42]. The activity of Nrf2 is fundamental for the maintenance of redox homeostasis because this transcription factor modulates the levels of key redox molecules by many signaling pathways [40,41,43,44]. In the present work, bergenin upregulated Nrf2 mRNA in the spinal cord and DRG of mice with DN, suggesting an effective control of redox imbalance at gene level, which agrees with the results found by Qiao et al. [26].

Among the numerous molecules whose synthesis is modulated by Nrf2, glutathione stands out as the most important molecule of the antioxidant defense system [38,45]. This is illustrated by the fact that the depletion of glutathione makes cells highly susceptible to oxidative injury [38]. When intracellular glucose levels are high, there is an upregulation of the polyol pathway, which ultimately leads to a decreased availability of glutathione [32], a critical factor for diabetic complications. Herein, bergenin treatment raised the *Gpx* mRNA levels in nervous tissues of neuropathic mice. These data are in agreement with previous studies carried out with bergenin [24,27] and show that the Nrf2 pathway is upregulated and functionally activated by this compound. Highlighting the relevance of the present data, treatments that increase glutathione levels, like bergenin, have been reported as effective strategies in the treatment of painful DN under experimental conditions [46,47].

Along with its antioxidant properties, glutathione prevents cytokine increase caused by hyperglycemia [48], which might be an additional protective mechanism, considering the well- established role of cytokines in maintaining painful DN. TNF-α and IL-1β are classically related to the development and maintenance of both inflammatory and neuropathic pain, as shown by extensive data obtained from both pre-clinical and clinical studies [49,50,51], and have thus been investigated here. The treatment with bergenin effectively reduced the increased levels of TNF-α and IL-1β both in the sciatic nerve and in the spinal cord of neuropathic mice. These results agree with previous reports that bergenin diminishes the release of these cytokines under different inflammatory conditions [12,17,18,20,33,52]. Mechanistic hypotheses have been proposed to explain the roles of TNF-α and IL-1β in the pathophysiology of DN. High glucose levels activate the TNF-α converting enzyme, which cleaves the transmembrane inactive form of TNF-α, releasing its soluble active form [53]. TNF-α contributes to painful DN by increasing the expression of sodium channels and thus the sodium current in the DRG, leading nociceptive sensitization [54]. IL-1β also plays a crucial role in the onset of DN [44,55]. IL-1β increases both amplitude and duration of action potentials in DRG neurons, contributing to peripheral sensitization [56]. Moreover, persistent hyperglycemia results in the glycosylation of myelin protein, which changes the antigenicity of neurons, promoting activation of glial cells, leukocyte infiltration, and release of inflammatory cytokines, including TNF-α and IL-1β, leading to neuroinflammation [57]. Hence, the control of neuroinflammation must be taken into account in the treatment of DN, which can be achieved by shifting the balance between pro- and anti-inflammatory cytokines. Some of the cytokines known to counteract the effects of TNF-α and IL-1β are IL-10 and TGF-β [58].

Bergenin treatment increased TGF-β levels in the sciatic nerve and spinal cord of neuropathic mice. These results were discordant from those of Yang, Kan, and Wu [31], who demonstrated that bergenin improves diabetic nephropathy by reducing renal production of TGF-β in diabetic rats. Such discrepancy might be due to the complex and dual roles of this cytokine in different systems [59]. Whereas TGF-β activation has been associated with fibrosis, cancer, and other diseases, it also plays a central role in inflammatory process as an immunosuppressive agent [60]. The present data are corroborated by previous works showing that increased TGF-β levels are associated with beneficial outcomes in experimental painful neuropathic conditions, including DN [8,61]. This cytokine exerts multiple neuroprotective mechanisms that diminish neuroinflammation and neuropathic pain. It reduces the spinal inflammatory response to peripheral damage by inhibiting glial cells activation, important sources of inflammatory mediators during the spinal neuroinflammation [62]. Thus, the enhanced levels of TGF-β in the peripheral and central nervous system can contribute to the long-lasting antinociceptive effect of bergenin in mice with painful DN.

Diabetic neuropathy is a complex clinical condition influenced by the interaction of multiple factors [4]. Current treatments fail to promote satisfactory analgesia in neuropathic patients, which has negative impacts in their life quality [6,7]. In this work, the therapeutic effects of bergenin in the painful DN were evidenced. In experimental conditions, bergenin reduced the behavioral signs of painful neuropathy with greater efficacy than gabapentin, the gold standard drug for clinical control of diabetic neuropathic pain. The sustained therapeutic effect promoted by bergenin over time reveals its potential of inducing long-lasting analgesia in patients with painful DN. Moreover, the antinociceptive effect of bergenin was associated with a shift of the cytokine balance towards anti-inflammatory predominance and upregulation of antioxidant pathways, favoring the reestablishment of redox and immune homeostasis in the microenvironment of the nervous system. Taken along with previously published data, our results point to a favorable pharmacological profile of bergenin as a drug candidate in the treatment of painful DN.

## 4. Materials and Methods 

### 4.1. Animals

Male C57BL/6 mice (25–30 g) were obtained from the Animal Facilities of Institute Gonçalo Moniz (FIOCRUZ; Salvador, Brazil) and housed in a temperature-controlled room (22 ± 2 °C), under a 12:12 h light-dark cycle of artificial light with free access to food and water. Animal care and handling procedures were in strict accordance with the recommendations in the Guide for the Care and Use of Laboratory Animals of the National Institutes of Health, Brazilian College of Animal Experimentation, and International Association for the Study of Pain for the use of laboratory animals [63]. All the procedures were approved by the Institutional Animal Care and Use Committee (Ethics Committee for Animal Experimentation of Fundação Oswaldo Cruz (FIOCRUZ), permit number 015/2013 and 025/2017; approved on 23 December 2013 and 27 March 2018, respectively). Every effort was made to minimize the number of animals used and any discomfort. Behavioral tests were performed without knowing to which experimental group each mouse belonged. 

### 4.2. Bergenin

Bergenin was isolated from the stem bark of *Cenostigma* *gardnerianum* (Fabaceae) as previously performed by our research group [17]. Botanical material was harvested in Casa Nova, BA, Brazil (09° 09′ 43” S; 40° 58′ 15” W) in authorized areas by IBAMA (Brazilian Institute for the Environment and Natural Resources). A voucher specimen has been deposited at the Herbarium of Universidade Estadual de Feira de Santana, Bahia, Brazil (HUEFS 78424). Bergenin was obtained as a crystalline solid (m. p. 238-240 ºC) and was identified by spectroscopic data comparison. Its purity was determined by HPLC as 98% (254 nm). [α]20D -37.0o, (c 0.20, MeOH), APCI/MS (neg.): 327 [(M-H)-]. IR ⭨ Max (cm^-1^): 3423, 3389, 3247, 3204, 2951, 2896, 1701, 1613, 1464, 1348, 1335, 1234, 1092, 1071, 991, 858 and 765 (KBr). 1H NMR (300 MHz, CD3OD): δ 7.07 (s, H-9), 4.95 (d, J = 10.5 Hz, H-1), 3.35-4,09 (m, H-11-14); 3.90 (s, H-15), 13C NMR (75 MHz, CD3OD): δ 119.37 (C-5), 117.24 (C-10), 152.28 (C-6), 142.21 (C-8), 149.38 (C-7), 111.04 (C-9), 165.79 (C-1), 74.19 (C-4), 75.58 (C-11), 81.34 (C-3); 71.82 (C-12), 82.98 (C-13), 62.63 (C-14), 60.93 (C-15).

### 4.3. Mouse Model of STZ-Induced Diabetes

Diabetes was experimentally induced in C57BL/6 mice as previously described by Guimarães et al. [64]. This model is characterized by the damage of pancreatic islets, reduced insulin production, hyperglycemia, weight loss, polyuria, and renal failure. Intraperitoneal injections of streptozotocin (STZ; 80 mg/kg in citrate buffer, pH 4.5) were given once a day for 3 successive days. Control mice received citrate buffer in the same volume, route, and schedule. Glycemia was determined in blood samples from the tail vein using ACCU-CHEK^®^ glucose sticks. Mice with dosages above 250 mg/dL were considered diabetic.

### 4.4. Experimental Design

To assess the effect of bergenin treatment on painful DN, two protocols were performed. Firstly, a single intraperitoneal injection of bergenin (3.125–25 mg/kg) or vehicle (2% dimethyl sulfoxide, DMSO, in saline) was given and the nociceptive threshold was evaluated from 2 to 24 h post-treatment. This protocol aimed to determine the dose-response relationship and the time of effect duration of the compound, allowing the selection of one dose to be used in the following experiments. The second protocol evaluated the effect of multiple administrations of bergenin (25 mg/kg) or vehicle (2% DMSO in saline) during 14 days, with two daily assessments of the threshold, aiming of determine the antinociceptive effect in a continuous treatment. Gabapentin (40 mg/kg, p.o.) twice a day for 14 days was used as the gold standard drug [65]. To exclude the possibility of movement impairment induced by bergenin, the motor capacity of mice was evaluated in the rota-rod test. At the end of the experimental period, nervous tissues samples were obtained for estimation of MDA and nitrite, evaluation of gene expression by qRT-PCR, and quantification of cytokine levels by ELISA.

### 4.5. Assessment of Sensory Neuropathy by Von Frey Filaments

Alteration of nociceptive thresholds is a major manifestation of DN, hence the nociceptive threshold to mechanical stimuli was measured with von Frey filaments (Stoelting; Chicago, IL, USA). Briefly, mice were placed in acrylic cages upon a wired grid floor, allowing their right hind paws to be touched with a series of filaments with logarithmically incremental stiffness. A positive response was characterized by the abrupt removal of the touched paw. The mechanical nociceptive threshold was calculated by the up-and-down method [66]. Values represent the filament weight (g) to which mice respond in 50% of presentations. The thresholds were evaluated daily for 3 days before the experimental procedures, to determine the baseline, and throughout the experimental period after the induction of the model. The development of painful DN was characterized by mechanical allodynia, indicated by the reduction of the paw nociceptive threshold.

### 4.6. The Rota-Rod Test for Assessment of Motor Impairment

The rota-rod apparatus (Insight; Ribeirão Preto, SP, Brazil) consists of a bar that rotates at a constant speed of 6 rpm and mice need to walk forwards to avoid falling from it. The animals were selected previously by eliminating those that did not remain on the bar for two consecutive periods of 120 s. Mice received bergenin (25 mg/kg, i.p.), vehicle (2% DMSO in saline), or the reference drug diazepam (10 mg/kg, i.p.) and, 40 min afterward, were placed on a rotating rod. The resistance to falling was measured up to 120 s [67].

### 4.7. In Vitro Assays: Cytotoxicity and Nitrite Levels in Macrophage Cultures

Initially, the cytotoxicity of bergenin to J774 macrophages was determined [68]. Murine macrophage-like cell line J774, kindly provided by Dr. Patricia S. T. Veras (Goncalo Moniz Institute, Fiocruz/BA), were seeded in 96-well plates at a cell density of 2 × 10^5^ cells/well in Dulbecco’s modified Eagle medium (DMEM; Life Technologies, GIBCO-BRL, Gaithersburg, MD, USA) supplemented with 10% fetal bovine serum (FBS; GIBCO), and gentamycin (50 μg/mL; Novafarma, Anápolis, GO, Brazil) and incubated at 37 °C and 5% CO_2_ for 2 h. Bergenin (3.125–200 μM) was then added in three replicates per concentration and plates were incubated for 72 h. Gentian violet (Synth, São Paulo, Brazil) at 10 μM was used as positive control. Alamar Blue (20 μL/well; Invitrogen, Carlsbad, CA) was added to the plates; after 12 h colorimetric readings were performed at 570 and 600 nm.

Next, the effect of non-cytotoxic concentrations of bergenin on nitrite levels, indicative of NO production by stimulated macrophages, was evaluated in triplicates as previously described [68]. J775 macrophages were seeded in 96-well tissue culture plates at 2 × 10^5^ cells/well in DMEM medium supplemented with 10% FBS and gentamycin (50 μg/mL) and incubated at 37 °C and 5% CO_2_ for 2 h. Cells were then stimulated with LPS (500 ng/mL, Sigma) and IFN-γ (5 ng/mL; Sigma) in the presence of bergenin (6.25–100 μM), vehicle (2% DMSO in saline), or dexamethasone (40 μM, reference drug). Unstimulated cells were used as negative control. After 24 h of incubation at 37 °C and 5% CO_2_, supernatants were collected and kept at −80 °C until quantification, which was done by the Griess method [69].

### 4.8. Estimation of Lipid Peroxidation and Nitrite in Nervous Tissues

At the end of the experimental period, spinal cord segments (L4–L5) and sciatic nerves were collected. Nervous tissues were rinsed with ice-cold saline and homogenized in chilled phosphate buffer (pH 7.4), then used to determine lipid peroxidation and nitrite estimation in triplicates [8]. The MDA content was assayed in the form of thiobarbituric acid-reactive substances (TBARS) [70]. Briefly, 0.5 mL of homogenate and 0.5 mL of Tris-HCl were incubated at 37 °C for 2 h. After incubation, 1 mL of trichloroacetic acid at 10% was added and centrifuged at 1000 g for 10 min. To 1 mL of supernatant, 1 mL of 0.67% thiobarbituric acid was added and the tubes were kept in boiling water for 10 min. After cooling, 1 mL double distilled water was added and absorbance was measured at 532 nm. TBARS were quantified using an extinction coefficient of 1.56 × 10^5^ M^−1^ cm^−1^ and were expressed as nmol of malondialdehyde per mg of protein. Nitrite, an indicator of nitric oxide production, was estimated in nervous tissues homogenates by the Griess method [69]. Briefly, 500 μL of Griess reagent (1:1 solution of 1% sulphanilamide in 5% phosphoric acid and 0.1% napthaylamine diamine dihydrochloric acid in water) was added to 100 μL of homogenate, and absorbance was measured at 546 nm. Nitrite concentration in μg per mL was calculated using a standard curve for sodium nitrite.

### 4.9. Real-Time PCR

The transcription of inducible nitric oxide synthase (*iNOS*), glutathione peroxidase (*Gpx*), and nuclear factor erythroid 2–related factor 2 (*Nrf2*) genes was evaluated by qRT-PCR in dorsal root ganglia (DRG) and spinal cord of mice, as previously described [8]. Total RNA was extracted from L4 and L5 DRG and spinal cord segments (L4-L5) with TRIzol reagent (Invitrogen, Carlsbad, CA, USA) and its concentration was determined by photometric measurement. A High-Capacity cDNA Reverse Transcription Kit (Applied Biosystems, Foster City, CA, USA) was used in the synthesis of cDNA from 1 μg of RNA, according to the manufacturer’s recommendations. Synthesis of cDNA and RNA expression analysis was performed by real-time PCR using TaqMan Gene Expression Assay for *iNOS* (Mm00440502_m1), *Gpx* (Mm00492427_m1) and *Nrf2* (Mm00477784_m1). A no-template control (NTC) and no-reverse transcription controls (No–RT) were included. All reactions were run in duplicate on an ABI7500 Sequence Detection System (Applied Biosystems) under standard thermal cycling conditions. The mean Ct (cycle threshold) values from duplicate measurements were used to calculate expression of the target gene, with normalization to an internal control—*Gapdh* (Mm99999915_g1).

### 4.10. Quantification of Cytokines Levels by ELISA

Cytokines were quantified in triplicates in the sciatic nerve and in the spinal cord of mice as previously described [71]. Tissue proteins were extracted from 100 mg tissue/mL phosphate buffered saline (PBS) to which 0.4 M NaCl, 0.05% Tween 20 and protease inhibitors (0.1 mM PMSF, 0.1 mM benzethonium chloride, 10 mM EDTA, and 20 KI aprotinin A/100 mL) were added. The samples were centrifuged for 10 min at 3000× *g* and the supernatant was frozen at −80 °C for later quantification. Tumor necrosis factor-α (TNF-α), interleukin-1β (IL-1β), and transforming growth factor- β (TGF-β) levels were determined using commercially available immunoassay ELISA kits for mice (R&D System; Minneapolis, MN, USA), according to the manufacturer’s instructions. Results were expressed as picograms of cytokine per milligram of protein.

### 4.11. Statistical Analysis

Data was presented as means ± SD of measurements made on six to eight mice per group. Comparisons between three or more treatments were made using one-way ANOVA with Tukey’s post-hoc test. For repeated measures (nociceptive threshold data), comparisons between groups were made by two-way ANOVA with Bonferroni’s post-hoc test. The factors analyzed were treatments, time and treatment–time interaction. Data were analyzed using GraphPad Prism 8 computer software (GraphPad, San Diego, CA, USA). Statistical differences were considered to be significant at *p* < 0.05.

## Abbreviations

AGE	Advanced glycation end product
BER	Bergenin
CTRL	Control
DEXA	Dexamethasone
DMEM	Dulbecco’s modified Eagle medium
DMSO	Dimethyl sulfoxide
DN	Diabetic neuropathy
DRG	Dorsal root ganglia
ELISA	Enzyme-linked immunosorbent assay
FBS	Fetal bovine serum
GABA	Gabapentin
Gpx	Glutathione peroxidase
GV	Gentian violet
IFN-γ	Interferon-γ
IL-1β	Interleukin-1β
iNOS	Inducible nitric oxide synthase
LPS	Lipopolysaccharide
MDA	Malondialdehyde
NF-κB	Nuclear factor kappa-light-chain-enhancer of activated B cells
NO	Nitric oxide
Nrf2	Nuclear factor erythroid 2-related factor 2
PBS	Phosphate buffered saline
ROS	Reactive oxygen species
RT-qPCR	Real-time quantitative polymerase chain reaction
STZ	Streptozotocin
TBARS	Thiobarbituric acid-reactive substances
TGF-β	Transforming growth factor-β
TNF-α	Tumor necrosis factor-α
VEH	Vehicle

## References

[1] Chaturvedi N. (2007). The burden of diabetes and its complications: Trends and implications for intervention. Diabetes Res. Clin. Pract..

[2] Ogurtsova K., da Rocha Fernandes J.D., Huang Y., Linnenkamp U., Guariguata L., Cho N.H., Cavan D., Shaw J.E., Makaroff L.E. (2017). IDF Diabetes Atlas: Global estimates for the prevalence of diabetes for 2015 and 2040. Diabetes Res. Clin. Pract..

[3] Tesfaye S., Boulton A.J., Dyck P.J., Freeman R., Horowitz M., Kempler P., Lauria G., Malik R.A., Spallone V., Vinik A. (2010). Diabetic neuropathies: Update on definitions, diagnostic criteria, estimation of severity, and treatments. Diabetes Care.

[4] Albers J.W., Pop-Busui R. (2014). Diabetic neuropathy: Mechanisms, emerging treatments, and subtypes. Curr. Neurol. Neurosci. Rep..

[5] Tesfaye S. (2011). Recent advances in the management of diabetic distal symmetrical polyneuropathy. J. Diabetes Investig..

[6] Khdour M.R. (2020). Treatment of diabetic peripheral neuropathy: A review. J. Pharm. Pharmacol..

[7] Waldfogel J.M., Nesbit S.A., Dy S.M., Sharma R., Zhang A., Wilson L.M., Bennett W.L., Yeh H.C., Chelladurai Y., Feldman D. (2017). Pharmacotherapy for diabetic peripheral neuropathy pain and quality of life: A systematic review. Neurology.

[8] Evangelista A.F., Vannier-Santos M.A., de Assis Silva G.S., Silva D.N., Juiz P.J.L., Nonaka C.K.V., Dos Santos R.R., Soares M.B.P., Villarreal C.F. (2018). Bone marrow-derived mesenchymal stem/stromal cells reverse the sensorial diabetic neuropathy via modulation of spinal neuroinflammatory cascades. J. Neuroinflammation.

[9] Galuppo M., Giacoppo S., Bramanti P., Mazzon E. (2014). Use of natural compounds in the management of diabetic peripheral neuropathy. Molecules.

[10] Abe K., Sakai K., Uchida M. (1980). Effects of bergenin on experimental ulcers—Prevention of stress induced ulcers in rats. Gen. Pharmacol. Vasc. Syst..

[11] Lim H.-K., Kim H.-S., Choi H.-S., Oh S., Choi J. (2000). Hepatoprotective effects of bergenin, a major constituent of Mallotus japonicus, on carbon tetrachloride-intoxicated rats. J. Ethnopharmacol..

[12] Xiang S., Chen K., Xu L., Wang T., Guo C. (2020). Bergenin Exerts Hepatoprotective Effects by Inhibiting the Release of Inflammatory Factors, Apoptosis and Autophagy via the PPAR-gamma Pathway. Drug Des. Dev. Ther..

[13] Shi X., Xu M., Luo K., Huang W., Yu H., Zhou T. (2019). Anticancer activity of bergenin against cervical cancer cells involves apoptosis, cell cycle arrest, inhibition of cell migration and the STAT3 signalling pathway. Exp. Ther. Med..

[14] Barai P., Raval N., Acharya S., Borisa A., Bhatt H., Acharya N. (2019). Neuroprotective effects of bergenin in Alzheimer’s disease: Investigation through molecular docking, in vitro and in vivo studies. Behav. Brain Res..

[15] Ji Y., Wang D., Zhang B., Lu H. (2019). Bergenin Ameliorates MPTP-Induced Parkinson’s Disease by Activating PI3K/Akt Signaling Pathway. J. Alzheimer’s Dis..

[16] Kumar R., Patel D.K., Prasad S.K., Laloo D., Krishnamurthy S., Hemalatha S. (2012). Type 2 antidiabetic activity of bergenin from the roots of Caesalpinia digyna Rottler. Fitoterapia.

[17] de Oliveira C.M., Nonato F.R., de Lima F.O., Couto R.D., David J.P., David J.M., Soares M.B., Villarreal C.F. (2011). Antinociceptive properties of bergenin. J. Nat. Prod..

[18] Gao X.J., Guo M.Y., Zhang Z.C., Wang T.C., Cao Y.G., Zhang N.S. (2015). Bergenin Plays an Anti-Inflammatory Role via the Modulation of MAPK and NF-kappaB Signaling Pathways in a Mouse Model of LPS-Induced Mastitis. Inflammation.

[19] Kumar S., Sharma C., Kaushik S.R., Kulshreshtha A., Chaturvedi S., Nanda R.K., Bhaskar A., Chattopadhyay D., Das G., Dwivedi V.P. (2019). The phytochemical bergenin as an adjunct immunotherapy for tuberculosis in mice. J. Biol. Chem..

[20] Lopes de Oliveira G.A., Alarcon de la Lastra C., Rosillo M.A., Castejon Martinez M.L., Sanchez-Hidalgo M., Rolim Medeiros J.V., Villegas I. (2019). Preventive effect of bergenin against the development of TNBS-induced acute colitis in rats is associated with inflammatory mediators inhibition and NLRP3/ASC inflammasome signaling pathways. Chem. Biol. Interact..

[21] Ren X., Ma S., Wang J., Tian S., Fu X., Liu X., Li Z., Zhao B., Wang X. (2016). Comparative effects of dexamethasone and bergenin on chronic bronchitis and their anti-inflammatory mechanisms based on NMR metabolomics. Mol. Biosyst..

[22] Zhang C., Zhao B., Zhang C., Qiu M., Ma S., Jin X., Shao Y., Wang M., Wang X. (2019). Mechanisms of bergenin treatment on chronic bronchitis analyzed by liquid chromatography-tandem mass spectrometry based on metabolomics. Biomed. Pharm..

[23] Alves C.Q., David J.M., David J.P., Villareal C.F., Soares M.B.P., Queiroz L.P.D., Aguiar R.M. (2012). Flavonoids and other bioactive phenolics isolated from Cenostigma macrophyllum (Leguminosae). Química Nova.

[24] de Oliveira G.A.L., da Silva Oliveira G.L., Nicolau L.A.D., Mafud A.C., Batista L.F., Mascarenhas Y.P., de Sousa L.K.M., David J.M., Pinto L.S., Alves C.Q. (2017). Bergenin from Peltophorum dubium: Isolation, Characterization, and Antioxidant Activities in Non-Biological Systems and Erythrocytes. Med. Chem..

[25] Qi Q., Dong Z., Sun Y., Li S., Zhao Z. (2018). Protective Effect of Bergenin against Cyclophosphamide-Induced Immunosuppression by Immunomodulatory Effect and Antioxidation in Balb/c Mice. Molecules.

[26] Qiao S., Liu R., Lv C., Miao Y., Yue M., Tao Y., Wei Z., Xia Y., Dai Y. (2019). Bergenin impedes the generation of extracellular matrix in glomerular mesangial cells and ameliorates diabetic nephropathy in mice by inhibiting oxidative stress via the mTOR/beta-TrcP/Nrf2 pathway. Free Radic. Biol. Med..

[27] Yun J., Lee Y., Yun K., Oh S. (2015). Bergenin decreases the morphine-induced physical dependence via antioxidative activity in mice. Arch. Pharmacal Res..

[28] Callaghan B.C., Cheng H.T., Stables C.L., Smith A.L., Feldman E.L. (2012). Diabetic neuropathy: Clinical manifestations and current treatments. Lancet Neurol..

[29] Lenzen S. (2008). The mechanisms of alloxan- and streptozotocin-induced diabetes. Diabetologia.

[30] Rakieten N., Rakieten M.L., Nadkarni M.V. (1963). Studies on the diabetogenic action of streptozotocin (NSC-37917). Cancer Chemother Rep..

[31] Yang J., Kan M., Wu G.Y. (2016). Bergenin ameliorates diabetic nephropathy in rats via suppressing renal inflammation and TGF-beta1-Smads pathway. Immunopharmacol. Immunotoxicol..

[32] Brownlee M. (2005). The pathobiology of diabetic complications: A unifying mechanism. Diabetes.

[33] Wang K., Li Y.F., Lv Q., Li X.M., Dai Y., Wei Z.F. (2017). Bergenin, Acting as an Agonist of PPARgamma, Ameliorates Experimental Colitis in Mice through Improving Expression of SIRT1, and Therefore Inhibiting NF-kappaB-Mediated Macrophage Activation. Front. Pharmacol..

[34] Lee C.H., Kim H.J., Lee Y.S., Kang G.M., Lim H.S., Lee S.H., Song D.K., Kwon O., Hwang I., Son M. (2018). Hypothalamic Macrophage Inducible Nitric Oxide Synthase Mediates Obesity-Associated Hypothalamic Inflammation. Cell Rep..

[35] Schmidtko A. (2015). Nitric oxide-mediated pain processing in the spinal cord. Handb. Exp. Pharmacol..

[36] Staunton C.A., Barrett-Jolley R., Djouhri L., Thippeswamy T. (2018). Inducible nitric oxide synthase inhibition by 1400W limits pain hypersensitivity in a neuropathic pain rat model. Exp. Physiol..

[37] Ahlawat A., Sharma S. (2018). A new promising simultaneous approach for attenuating type II diabetes mellitus induced neuropathic pain in rats: iNOS inhibition and neuroregeneration. Eur. J. Pharmacol..

[38] Vincent A.M., Russell J.W., Low P., Feldman E.L. (2004). Oxidative stress in the pathogenesis of diabetic neuropathy. Endocr. Rev..

[39] Cellek S., Qu W., Schmidt A.M., Moncada S. (2004). Synergistic action of advanced glycation end products and endogenous nitric oxide leads to neuronal apoptosis in vitro: A new insight into selective nitrergic neuropathy in diabetes. Diabetologia.

[40] Ahmed S.M., Luo L., Namani A., Wang X.J., Tang X. (2017). Nrf2 signaling pathway: Pivotal roles in inflammation. Biochim. Biophys. Acta (BBA)-Mol. Basis Dis..

[41] Bellezza I., Giambanco I., Minelli A., Donato R. (2018). Nrf2-Keap1 signaling in oxidative and reductive stress. Biochim. Biophys. Acta (BBA)-Mol. Cell Res..

[42] Strom J., Xu B., Tian X., Chen Q.M. (2016). Nrf2 protects mitochondrial decay by oxidative stress. FASEB J..

[43] Ganesh Yerra V., Negi G., Sharma S.S., Kumar A. (2013). Potential therapeutic effects of the simultaneous targeting of the Nrf2 and NF-kappaB pathways in diabetic neuropathy. Redox Biol..

[44] Li J., Hu X., Liang F., Liu J., Zhou H., Liu J., Wang H., Tang H. (2019). Therapeutic effects of moxibustion simultaneously targeting Nrf2 and NF-kappaB in diabetic peripheral neuropathy. Appl. Biochem. Biotechnol..

[45] Lu S.C. (2013). Glutathione synthesis. Biochim. Biophys. Acta.

[46] Erbas O., Taskiran D., Oltulu F., Yavasoglu A., Bora S., Bilge O., Cinar B.P., Peker G. (2017). Oxytocin provides protection against diabetic polyneuropathy in rats. Neurol. Res..

[47] Wang Y., Chen Z., Ye R., He Y., Li Y., Qiu X. (2013). Protective effect of Jiaweibugan decoction against diabetic peripheral neuropathy. Neural Regen. Res..

[48] Esposito K., Nappo F., Marfella R., Giugliano G., Giugliano F., Ciotola M., Quagliaro L., Ceriello A., Giugliano D. (2002). Inflammatory cytokine concentrations are acutely increased by hyperglycemia in humans: Role of oxidative stress. Circulation.

[49] Hung A.L., Lim M., Doshi T.L. (2017). Targeting cytokines for treatment of neuropathic pain. Scand. J. Pain.

[50] Safieh-Garabedian B., Nomikos M., Saade N. (2019). Targeting inflammatory components in neuropathic pain: The analgesic effect of thymulin related peptide. Neurosci. Lett..

[51] Cohen S.P., Mao J. (2014). Neuropathic pain: Mechanisms and their clinical implications. BMJ.

[52] Yang S., Yu Z., Wang L., Yuan T., Wang X., Zhang X., Wang J., Lv Y., Du G. (2017). The natural product bergenin ameliorates lipopolysaccharide-induced acute lung injury by inhibiting NF-kappaB activition. J. Ethnopharmacol..

[53] Reddy A.B., Ramana K.V., Srivastava S., Bhatnagar A., Srivastava S.K. (2009). Aldose reductase regulates high glucose-induced ectodomain shedding of tumor necrosis factor (TNF)-alpha via protein kinase C-delta and TNF-alpha converting enzyme in vascular smooth muscle cells. Endocrinology.

[54] de Macedo F.H.P., Aires R.D., Fonseca E.G., Ferreira R.C.M., Machado D.P.D., Chen L., Zhang F.X., Souza I.A., Lemos V.S., Romero T.R.L. (2019). TNF-alpha mediated upregulation of NaV1.7 currents in rat dorsal root ganglion neurons is independent of CRMP2 SUMOylation. Mol. Brain.

[55] Chessell I.P., Hatcher J.P., Bountra C., Michel A.D., Hughes J.P., Green P., Egerton J., Murfin M., Richardson J., Peck W.L. (2005). Disruption of the P2X7 purinoceptor gene abolishes chronic inflammatory and neuropathic pain. Pain.

[56] Noh M.C., Stemkowski P.L., Smith P.A. (2019). Long-term actions of interleukin-1beta on K(+), Na(+) and Ca(2+) channel currents in small, IB4-positive dorsal root ganglion neurons; possible relevance to the etiology of neuropathic pain. J. Neuroimmunol..

[57] Sandireddy R., Yerra V.G., Areti A., Komirishetty P., Kumar A. (2014). Neuroinflammation and oxidative stress in diabetic neuropathy: Futuristic strategies based on these targets. Int. J. Endocrinol..

[58] Benveniste E.N., Tang L.P., Law R.M. (1995). Differential regulation of astrocyte TNF-alpha expression by the cytokines TGF-beta, IL-6 and IL-10. Int. J. Dev. Neurosci..

[59] Tzavlaki K., Moustakas A. (2020). TGF-beta Signaling. Biomolecules.

[60] Blobe G.C., Schiemann W.P., Lodish H.F. (2000). Role of transforming growth factor beta in human disease. N. Engl. J. Med..

[61] Chen N.F., Huang S.Y., Lu C.H., Chen C.L., Feng C.W., Chen C.H., Hung H.C., Lin Y.Y., Sung P.J., Sung C.S. (2014). Flexibilide obtained from cultured soft coral has anti-neuroinflammatory and analgesic effects through the upregulation of spinal transforming growth factor-beta1 in neuropathic rats. Mar. Drugs.

[62] Echeverry S., Shi X.Q., Haw A., Liu H., Zhang Z.W., Zhang J. (2009). Transforming growth factor-beta1 impairs neuropathic pain through pleiotropic effects. Mol. Pain.

[63] Zimmermann M. (1983). Ethical guidelines for investigations of experimental pain in conscious animals. Pain.

[64] Guimarães E.T., Cruz Gda S., Almeida T.F., Souza B.S., Kaneto C.M., Vasconcelos J.F., Santos W.L., Santos R.R., Villarreal C.F., Soares M.B. (2013). Transplantation of stem cells obtained from murine dental pulp improves pancreatic damage, renal function, and painful diabetic neuropathy in diabetic type 1 mouse model. Cell Transplant..

[65] Reda H.M., Zaitone S.A., Moustafa Y.M. (2016). Effect of levetiracetam versus gabapentin on peripheral neuropathy and sciatic degeneration in streptozotocin-diabetic mice: Influence on spinal microglia and astrocytes. Eur. J. Pharmacol..

[66] Dixon W.J. (1965). The Up-and-Down Method for Small Samples. J. Am. Stat. Assoc..

[67] Leite dos Santos G.G., Casais e Silva L.L., Pereira Soares M.B., Villarreal C.F. (2012). Antinociceptive properties of Micrurus lemniscatus venom. Toxicon.

[68] Espirito-Santo R.F., Meira C.S., Costa R.D.S., Souza Filho O.P., Evangelista A.F., Trossini G.H.G., Ferreira G.M., Velozo E.D.S., Villarreal C.F., Pereira Soares M.B. (2017). The anti-inflammatory and immunomodulatory potential of braylin: Pharmacological properties and mechanisms by in silico, in vitro and in vivo approaches. PLoS ONE.

[69] Green L.C., Wagner D.A., Glogowski J., Skipper P.L., Wishnok J.S., Tannenbaum S.R. (1982). Analysis of nitrate, nitrite, and [15N]nitrate in biological fluids. Anal. Biochem..

[70] Tiwari V., Kuhad A., Chopra K. (2009). Tocotrienol ameliorates behavioral and biochemical alterations in the rat model of alcoholic neuropathy. Pain.

[71] Gama K.B., Santos D.S., Evangelista A.F., Silva D.N., de Alcantara A.C., Dos Santos R.R., Soares M.B.P., Villarreal C.F. (2018). Conditioned Medium of Bone Marrow-Derived Mesenchymal Stromal Cells as a Therapeutic Approach to Neuropathic Pain: A Preclinical Evaluation. Stem Cells Int..

